# Case of Puerperal Convulsions, Treated with Chloroform

**Published:** 1862-02

**Authors:** Alfred Wharton

**Affiliations:** St. Paul, Minnesota


ART. V.—*Case of Puerperal Convulsions, treated with Chloroform, by* ALFRED WHARTON, M, D., *St. Paul, Minnesota.*

On the morning of November 15, 1861, at 2 A. M., I was summoned to see Mrs. H——, who, the messenger told me, was in labor with her first child, and had convulsions. Without further delay I hastened to the bed-side. I found Mrs. H. tossing about in bed, her eyes wide open, with a vacant, staring expression, giving no heed to various questions made to her by her friends. In a little while, slow jerking movements were noticed in her head, which gradually extended over the muscles of the arms, trunk, and lower extremities, until the whole body became violently convulsed.—Respiration was irregular and disordered, with a quantity of foam and mucus escaping from the mouth; the tongue protruded to one side, and the jaws tightly closed; pulse 110—feeble.

On making inquiry of the friends of the patient, I learned she was taken with labor pains about ten o'clock in the evening, and since one o'clock in the morning had five convulsions. On making examination I found the os uteri neither dilated or dilatable, and seeing no prospects of bringing on labor, I directed my attention to the convulsed condition of the patient, and determined on the exhibition of chloroform in hope of quieting her. I placed her immediately under the influence of the anæsthetic, in this her sixth and most painful convulsion; and no sooner had a slight influence been produced, when the severity of the attack subsided, and gradually disappeared. I continued its application, (never producing full anæsthesia) for at least three hours, when I gradually allowed her to return to consciousness, when her system showed signs of an approaching attack. The agent was re-applied, with a similar happy effect, and continued for over two hours, when it was discontinued without any sign of a return of the disorder.

In the course of a few hours—the os being dilated—I ruptured the membranes, when she speedily gave birth to a dead fœtus. The placenta came away nicely, and she had a rapid recovery.

It will be noticed that the only remedial agent made use of, was the anæsthetic, with the exception of iced applications to the head.

*Remarks.*—Many of our professional brethren are averse to the exhibition of chloroform in this terrible disease; still, all will admit, it matters but little what measures we employ, provided the desired end be accomplished, namely: the preservation of the life of the mother and child. If experience shall prove that chloroform is as safe, as it is efficient in these cases, what a victory we have gained in therapeutics over the much extolled plan of treatment, which has been in vogue for so many years, viz: blood-letting, blistering, calomel, and such like.

In all these cases the physician has but little time for meditation; he must act, and that promptly, for the happiness of a family and the life of his patient are at stake. He must call to mind, and decide in an instant, the treatment to be pursued, if he would rescue the patient from impending death.

It will give me great pleasure to contribute my mite, since every case reported, where the treatment has been successful, adds another link to the great chain of medical experience.

